# Influenza Immunization in Very-Low-Birth-Weight Infants: Epidemiology and Long-Term Outcomes

**DOI:** 10.3390/vaccines13010042

**Published:** 2025-01-07

**Authors:** Marie-Theres Dammann, Hannah Kraft, Guido Stichtenoth, Kathrin Hanke, Michael Zemlin, Janina Soler Wenglein, Isabell Ricklefs, Alexander Herz, Alexander Humberg, Dorothee Viemann, Geraldine Engels, Matthias Volkmar Kopp, Folke Brinkmann, Carsten Fortmann-Grote, Wolfgang Göpel, Egbert Herting, Christoph Härtel, Ingmar Fortmann, on behalf of the German Neonatal Network

**Affiliations:** 1 Department of Pediatrics, University Hospital of Lübeck, 23538 Lübeck, Germany; hannah.kraft@student.uni-luebeck.de (H.K.); guido.stichtenoth@uksh.de (G.S.); kathrin.hanke@uksh.de (K.H.); alexander.herz@uksh.de (A.H.); matthias.kopp@insel.ch (M.V.K.); folke.brinkmann@uksh.de (F.B.); wolfgang.goepel@uksh.de (W.G.); egbert.herting@uksh.de (E.H.); matsingmar.fortmann@uksh.de (I.F.); 2 Department of General Pediatrics and Neonatology, Saarland University, 66123 Homburg, Germany; michael.zemlin@uks.eu; 3 Department of Pediatrics, Protestant Hospital of the Bethel Foundation, Medical School and University Medical Center East Westphalia-Lippe, Bielefeld University, 33617 Bielefeld, Germany; janina.soler@uni-bielefeld.de; 4 Department of General Pediatrics, University Children’s Hospital Münster, 48149 Münster, Germany; alexander.humberg@ukmuenster.de; 5 Center for Infection Research, University of Würzburg, 97080 Würzburg, Germany; viemann_d@ukw.de; 6 Department of Pediatrics, University of Würzburg, 97080 Würzburg, Germany; engels_g@ukw.de (G.E.); haertel_c1@ukw.de (C.H.); 7 Cluster of Excellence RESIST (EXC 2155), Hannover Medical School, 30625 Hannover, Germany; 8 Airway Research Center North, German Center of Lung Research (DZL), 23562 Lübeck, Germany; 9 Department of Paediatric Respiratory Medicine, Inselspital, University Children`s Hospital of Bern, 3008 Bern, Switzerland; 10 Scientific Computing Unit, Department of Microbial Population Biology, Max-Plank-Institute for Evolutionary Biology, 24306 Plön, Germany; carsten.fortmann-grote@evolbio.mpg.de

**Keywords:** immunization, influenza, VLBWI, lung function

## Abstract

Background: Very-low-birth-weight infants (VLBWIs; birth weight < 1500 g) are at an increased risk of complicated influenza infection, which frequently includes pneumonia, encephalitis or even death. Data on influenza immunization and its outcome in VLBWIs are scarce. This study aimed to provide epidemiological data on influenza immunization for German VLBWIs and hypothesized that immunization would protect VLBWIs from infection-mediated neurodevelopmental impairment and preserves lung function at early school age. Methods: In this observational population-based German Neonatal Network (GNN) study, infants born between 2009 and 2015 were invited to partake in a 6-year follow-up investigation including lung function and developmental testing. Uni- and multivariate analyses were performed to evaluate the clinical characteristics and outcomes of influenza-immunized VLBWIs compared to non-immunized VLBWIs. Results: Influenza immunization was performed in 871 out of the 3358 VLBWIs (26%) with six-year follow-up. Immunized infants were characterized by a low gestational age and higher rates of morbidity, particularly bronchopulmonary dysplasia. Although early immunization showed no safety signals and had protective effects on the long-term risk of bronchitis (OR: 0.2; CI: 0.1–0.6; *p* = 0.002), most VLBWIs (88.0%) were unimmunized in their first influenza season. Conclusions: Influenza immunization was not associated with improved lung function (forced expiratory volume in one second and forced vital capacity) or a better neurocognitive outcome (intelligence quotient and strengths and difficulties questionnaire) at early school age. In Germany, only one quarter of 6-year-old VLBWIs were immunized against influenza, particularly those born <28 gestational weeks and/or BPD. Specific influenza immunization guidelines that define evidence-based recommendations are needed for this vulnerable group.

## 1. Introduction

Very-low-birth-weight infants (birth weight < 1500 g; VLBWIs) are at a high risk of respiratory tract infections, including infection with influenza virus or respiratory syncytial virus (RSV) [1]. With medical advances enabling the survival of smaller and more immature preterm infants, an increasing number of VLBWIs are experiencing long-term respiratory impairments due to lung immaturity and/or bronchopulmonary dysplasia (BPD) [2,3]. Influenza is a leading cause of lower respiratory tract infection, with an estimated 109.5 million cases of influenza virus infection in children under five years of age in 2018 [4]. The risk of severe disease progression and the long-term impairment of the lungs is particularly high in infants with chronic underlying conditions [1,5]. Prematurity is a known risk-factor for influenza-related hospital admission [6,7]. Persistent viral presence in the lungs can contribute to the development of chronic lung disease (CLD) [8,9,10]. The heightened susceptibility of VLBWIs to infections is attributed to distinct and immature features of the immune system and persists after primary hospital stay into infancy until school age [11]. However, data on the long-term impact of influenza infection in VLBWIs, including lung function, are lacking. Perinatal strategies such as antenatal corticosteroids, continuous positive airway pressure (CPAP), less invasive surfactant administration and caffeine are known to improve pulmonary outcomes in VLBWIs [12]. Secondary preventive strategies include active immunization, which represents a safe, effective, cost-efficient and immunogenic strategy.

Beyond its respiratory effects, influenza virus is neurotropic, with the central nervous system being the most common extrapulmonary site of influenza manifestation [13]. Influenza infection during a critical phase of neurodevelopment is believed to cause deviations in neurocognitive abilities [14,15,16,17,18]. It has been discussed whether childhood infections are associated with intelligence quotient (IQ) levels [19].

The German Standing Committee on Vaccination (STIKO) recommends seasonal immunizations against influenza for people at an increased risk of infection due to underlying medical conditions from the age of six months [20]. STIKO-defined underlying medical conditions include (1) airway hypersensitivity or lung function impairment, (2) chronic disorders of the cardiovascular system, liver, or kidney, (3) diabetes and other metabolic disorders, (4) neurologic or neuromuscular diseases and (5) impaired immune function. Infants should receive the same vaccine dose as adults, with two doses recommended for first-time vaccination, administered four weeks apart. For healthy children, influenza vaccination is not recommended, as complications are rare in this group [21]. To our knowledge, no epidemiological data are available on influenza immunization rates among VLBWIs in Germany, and international data remain limited. North American data, however, indicate that full influenza immunization coverage is highest among VLBWIs born at 23 to 33 weeks of gestation (47.7%), lowest among late preterm VLBWIs (34 to 36 weeks, 41.5%), and 44.7% in term infants [22]. Full coverage is defined as two to three doses (depending on the month of birth) by 19 months or four doses by 36 months, based on American immunization guidelines. The American Center for Disease Control and Prevention (CDC) recommends annual influenza vaccination, starting at six months of age regardless of preterm birth or underlying medical conditions [23].

Within the large GNN cohort of VLBWIs, we aimed to provide epidemiological data on influenza immunization. We hypothesized that preventing seasonal influenza through active immunization protects VLBWIs from neurodevelopmental and behavioral impairment (assessed by IQ and the Strengths and Difficulties Questionnaire [SDQ]) and lung function impairment (evaluated by forced expiratory volume in one second [FEV_1_] and forced vital capacity [FVC]) at early school age.

## 2. Patients and Methods

**Figure 1:**
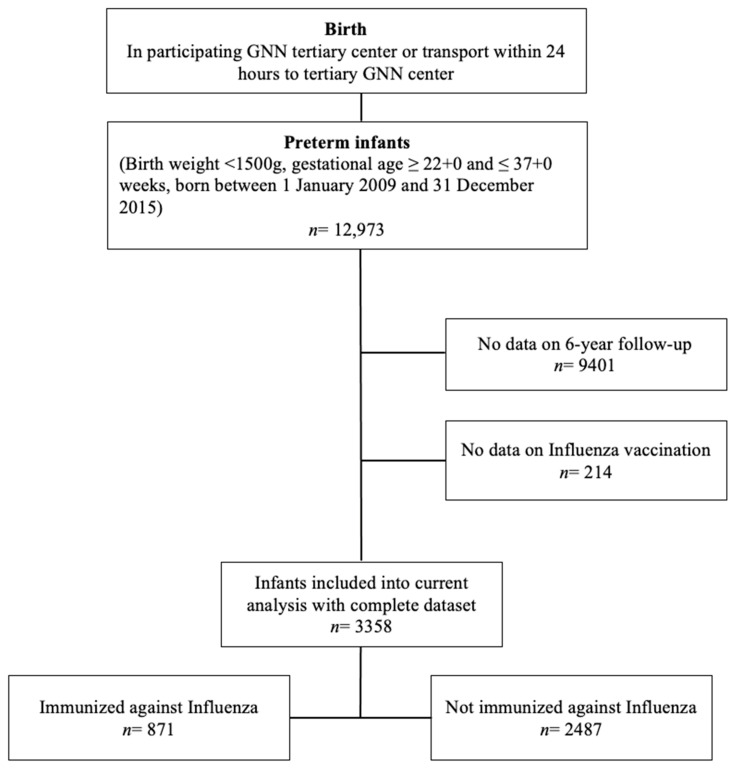
Flowchart of included and excluded VLBWIs within the GNN study cohort.

The German Neonatal Network (GNN; www.vlbw.de; accessed on 2 January 2025) is a population-based multicenter cohort study of VLBWIs in 68 neonatal intensive care units (NICU) across Germany. The data for the observational investigation were collected from VLBWIs born in 52 centers between 1 January 2009 and 31 December 2015. VLBWIs with a birth weight of <1500 g and a gestational age between 22 + 0 and 36 + 6 weeks met the inclusion criteria. VLBWIs with lethal malformations or without complete data sets were excluded from the current analysis (Figure 1). After obtaining informed written consent from the parents or legal guardians, the VLBWIs’ pre- and neonatal characteristics, treatment and outcome were assessed based on clinical records at the participating GNN centers. Data were analyzed at the study center in Lübeck. A physician trained in neonatology, or a study nurse, monitored the data quality by performing annual on-site visits.

### 2.1. Six-Year Follow up

At the age of six years, children participating in the GNN were invited to attend a follow-up assessment organized by the study office, where they were examined by a trained neonatologist and two study nurses. We selected five to six years of age for the follow-up assessment primarily for pragmatic reasons, aiming for high follow-up rates by choosing a relatively early time point, while ruling out an assessment at two years due to the unreliability of developmental tests at that age. The study team coordinated with the birth clinic to schedule potential on-site follow-up examination dates. The database of potential candidates was randomly searched, with a focus on VLBWIs born before 28 weeks of gestation. The invitation procedure was standardized across all participating centers. A single GNN follow-up team, blinded to any interventions or complications experienced during the initial NICU stay, conducted the assessments on-site. To ensure consistent results, the same equipment and instruments were used for all assessments by the GNN team. Standardized tests were performed, such as the Movement Assessment Battery for VLBWIs, the movement assessment battery for children, and the Wechsler Preschool and Primary Scale of Intelligence (Third Edition; WPPSI I–III), to assess their motor and cognitive development. A hearing test, visual screening and spirometry were performed. Information about the social background, medical history and general behavior of the parents or legal guardians was collected using standardized questionnaires. Data from the immunization record, including all immunizations performed until follow-up, were collected and entered into the database.

Spirometry was conducted using an EasyOne spirometer (NDD Medizintechnik AG, Zürich, Switzerland) by the study team’s physician, who assessed the quality of each spirometry test by evaluating the volume–time and flow–volume curves. The EasyOne spirometer program also provided built-in technical validation of the volume loops, ensuring that only results of adequate quality were considered. FEV_1_ and FVC were measured in liters and analyzed as the percentages of predicted values based on a European reference population [24]. Additionally, FEV_1_ and FVC z-scores were calculated according to the Global Lung Function Initiative guidelines [25]. The parental questionnaires, part of the GNN follow-up, included questions about the frequency of bronchitis in the past year (secondary respiratory outcome parameter).

Research question: The present study aimed to present epidemiological population-based data on influenza immunization in German VLBWIs and the associated clinical characteristics. We hypothesized that immunization would protect preterm infants from long-term morbidities in terms of lung function and neurodevelopmental outcome.

Definitions: Gestational age was calculated from the best obstetric estimate obtained on early prenatal ultrasound and obstetric examination [26]. VLBWIs were defined as infants with a birth weight under 1500 g. Small for gestational age (SGA) was defined as a birth weight below the 10th percentile for gestational age according to the sex-specific standards for birth weight by gestational age in Germany [27]. Blood-culture-proven sepsis was defined as clinical sepsis with proof of a causative pathogen [28]. BPD was defined by considering two grades [29]. Severe BPD was diagnosed when the need for supplemental oxygen or ventilatory support at 36 weeks of postmenstrual age was present. Mild BPD was diagnosed when supplemental oxygen or ventilatory support at 28 days of age was required but did not persist up until 36 weeks of postmenstrual age. Intraventricular hemorrhage (IVH) was defined as a bleed into the brain’s ventricles [30]. Grade I IVH was defined as a hemorrhage limited to the germinal matrix, and is also referred to as a subependymal bleed. Grade II IVH was defined as a bleed into the lumen of the ventricle, filling up to half of the lumen. Grade III IVH was defined as an intraventricular bleed filling the entire ventricle and leading to the dilation of the ventricle. Grade IV IVH was defined as a posthemorrhagic infarction. Severe complications were defined as 1. grade III or IV IVH, 2. periventricular leukomalacia, 3. retinopathy of prematurity requiring surgical intervention, 4. persistent ductus arteriosus requiring surgical intervention, 5. focal intestinal perforation requiring surgical intervention, or 6. ventriculoperitoneal shunt surgery.

Heptavalent immunization encompasses immunization against diphtheria, tetanus, pertussis, poliomyelitis, haemophilus influenzae type B, hepatitis B and pneumococcus. Influenza immunization was defined as having received at least one dose of influenza immunization. Usual care was an adult dose of influenza vaccine. During the 2009 H1N1 pandemic, the Paul Ehrlich Institute for Drug Safety recommended the use of half an adult dose (0.25 mL) for the pandemic influenza vaccine in children. Discharge into influenza season was defined as discharge from primary hospital care between December and March. The start of the influenza season was derived from the RKI’s report on the epidemiology of Influenza in Germany for the seasons 2009/10 until 2016/17 [31]. The respective dates for the start of each season were 12 October 2009, 6 December 2010, 6 February 2012, 10 December 2012, 17 February 2014, 5 January 2015, and 11 January 2016. The start of a season was defined as the timepoint at which influenza viruses were detected in every 5th patient’s sample by the National Reference Center for Influenza Viruses.

An infant was considered to have received an early vaccination if he or she was at least six months old—and therefore eligible for influenza vaccination—before the start of their first influenza season and had been vaccinated at least once by the end of the first month of that season.

### 2.2. Statistical Analysis

Data were analyzed using the IBM SPSS statistics version 29.0 [32]. Only complete datasets for all outcome parameters were included in the analysis (Figure 1). The global type 1 error level was set at *p* = 0.05 for every parameter. Univariate analyses were performed using the chi^2^ test, *t*-test or Mann–Whitney *U* test. To determine the possible associations between influenza immunization and the respiratory or neurocognitive outcome of VLBWIs, multivariate logistic and linear regression models were conducted. Therefore, we included known confounding variables, i.e., gestational age, sex, multiple birth, SGA status, BPD, severe complications (1. IVH grade III or IV, 2. periventricular leukomalacia, 3. surgeries for retinopathy of prematurity, 4. focal intestinal perforation, 5. persistent ductus arteriosus or 6. for a ventricular peritoneal shunt) and blood-culture-proven sepsis. It is important to note that FEV_1_ and FVC z-scores are already adjusted for gender and postnatal age. The odds ratios and 95% confidence intervals (CIs) were calculated to estimate the effect of an influenza immunization on the primary outcomes. To avoid the accumulation of alpha errors, Bonferroni corrections were performed for multivariate analyses to protect them from statistical type I errors. Alternatively, we matched the groups of immunized and non-immunized VLBWIs via Mahalanobis distance multidimensional modeling in order to reduce the selection bias within our observational data. Matching was based on the calculated Mahalanobis distance, including the gestational age, birth weight, gender, multiple birth, SGA and BPD. Each immunized VLBWI was matched to the nearest control with the smallest Mahalanobis distance, ensuring that matched pairs were as similar as possible across the covariates. Graphics were created with R Studio using version 2024.04.2+764 and R version 4.2.2 by Posit Software, PBC [33].

## 3. Results

Study cohort. During the observational period from 1 January 2009, until 31 December 2015, *n* = 12,973 VLBWIs were born in one of the 52 participating GNN centers (at that time) (Figure 1). We excluded *n* = 9401 VLBWIs from the current analysis, because no data on the 6-year follow-up were available. Information on influenza immunization was missing in *n* = 214 VLBWIs. The remaining *n* = 3358 VLBWIs were included in the analysis. The study cohort was stratified into VLBWIs that received at least one dose of influenza immunization (*n* = 871) and those who were never immunized against influenza until follow-up (*n* = 2487), corresponding to an immunization rate of 25.94%.

### 3.1. Clinical Characteristics of the Study Cohort

The baseline clinical characteristics are shown in Table 1. The study cohort displayed a median gestational age of 28.1 weeks (interquartile range [IQR] 26.3–29.7) and a median birth weight of 990 g (IQR 770–1240). Immunized VLBWIs had a lower birth weight (median 900 g vs. 1030 g) and gestational age (27.3 weeks vs. 28.4 weeks), respectively, than non-immunized infants. No differences were identified for multiple birth, SGA status, maternal descent and age at follow-up (median 5.75 years, IQR 5.41–6.0, *p* = 0.754). In our cohort, 23.7% of the infants were immunized against influenza but not RSV, whereas 55.9% were immunized against RSV but not influenza.

### 3.2. Epidemiology of Influenza Immunization in German VLBWIs

**Figure 2:**
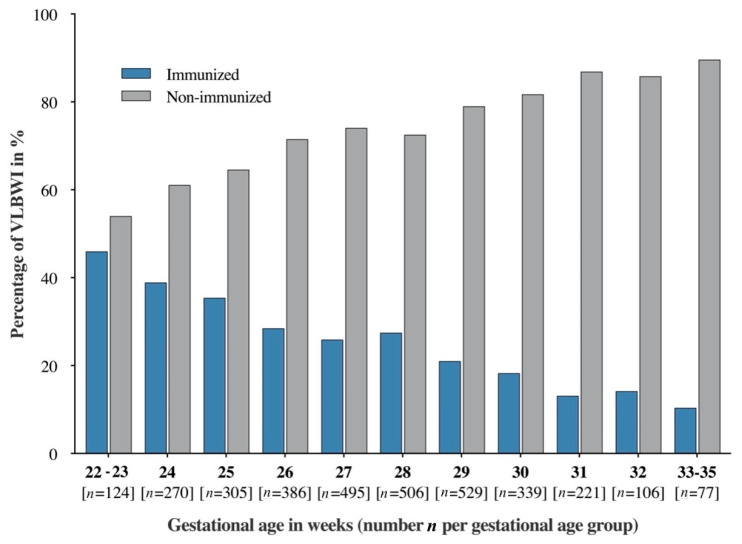
Immunization rates in VLBWIs by gestational age. Percentages of VLBWIs who are immunized (blue) versus non-immunized (grey) across gestational age groups. “Immunized” refers to infants who have received at least one dose of the influenza vaccine. The overall vaccination rate in VLBWIs was 25.9%. Among extremely-low-gestational-age neonates (ELGANs, <28 weeks), 32.2% were immunized, compared to 21.3% of very preterm VLBWIs (28–31 + 6 weeks) and 12.6% of moderate to late preterm VLBWIs (32–36 + 6 weeks). Immunization rates were highest in VLBWIs born at 22–23 weeks (45.9%) and in those with BPD (39.4%). Immunization followed seasonality, as 80% of all vaccinations were performed between October and December.

In total, 871 VLBWIs (25.94%) were immunized against influenza. Most VLBWIs received at least two vaccine doses (Figure S1). Notably, the majority of immunized VLBWIs received their first dose of influenza vaccine in their second year of life (the median age at first vaccination was 23 months, Figure S2). Only 23.6% of VLBWIs were immunized in the first year after discharge from primary hospital care, whereas 36.3% (17%, 11.3% and 8.4%) were vaccinated in the second year (third, fourth and fifth year after hospital discharge). Influenza immunization before hospital discharge was very rare (0.3%). In the GNN cohort, 49.1% of VLBWIs (*n* = 1650) were at least six months old before their first influenza season, making them eligible for immunization. However, only 12.0% (*n* = 198) of these eligible infants received the vaccine prior to that season. As shown in Figure 2, gestational age was negatively correlated with the influenza immunization rate. The highest immunization rates were observed among the most premature VLBWIs, i.e., in the age group of 22–23 gestational weeks (45.9%) and infants with BPD (39.4%). The immunization rates differed significantly across the federal states of Germany. In the eastern federal states of Germany (former German Democratic Republic; GDR), 30.6% of six-year-old VLBWIs were immunized against influenza, while the immunization rate in the western federal states was significantly lower, at 24.8% (*p* = 0.003).

### 3.3. Clinical Characteristics of Influenza Immunized VLBWI

**Figure 3:**
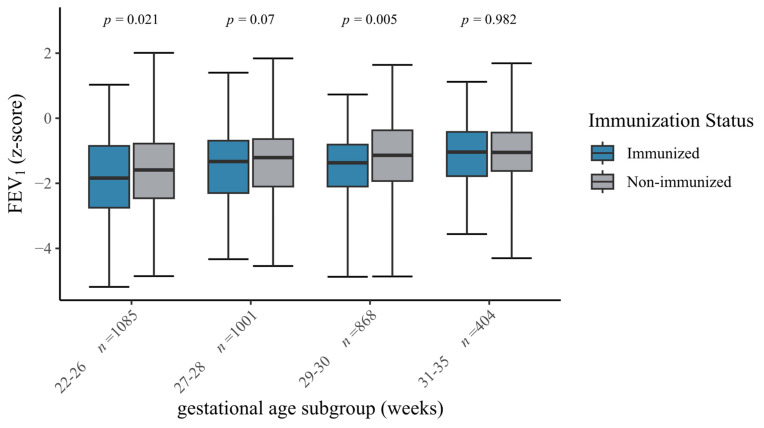
FEV_1_ z-scores of VLBWIs by gestational age and immunization status. The box plots show the forced expiratory volume in one second (FEV_1_) z-scores stratified to the gestational age subgroups and immunization status (influenza) at the six-year follow-up. The box indicates the median, 25th percentile and 75th percentile. The error bars indicate minimum and maximum values. *p*-Values are derived from the Mann–Whitney *U* test.

**Figure 4:**
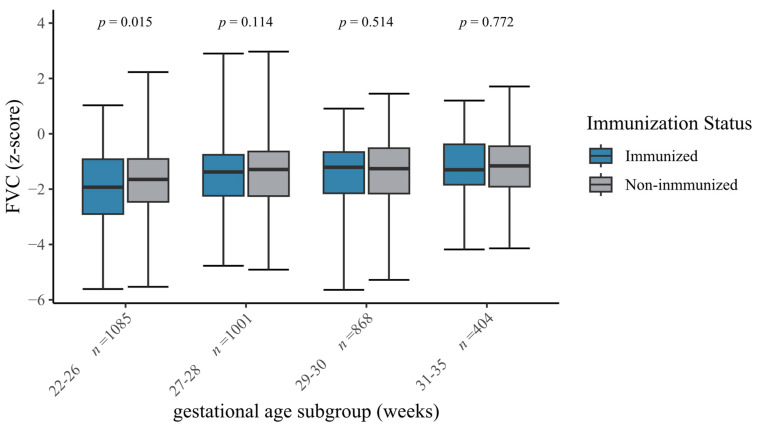
FVC z-scores of VLBWIs by gestational age and immunization status. The figure shows box plots of the forced vital capacity (FVC) z-scores stratified to the gestational age subgroups and immunization status at the six-year follow-up. The box indicates the median, 25th percentile and 75th percentile. The error bars indicate minimum and maximum values. *p*-Values are derived from the Mann–Whitney *U* test.

Infants with influenza immunization were characterized by higher rates of respiratory morbidity in the univariate analyses, such as BPD, bronchitis episodes, ventilation and impaired spirometry measures, when compared to non-immunized VLBWIs (Table 2, Figure 3 and Figure 4). Hence, immunized infants were more often passively immunized against RSV (76.3%, Table 1). In VLBWIs born at 22–26 and 29–30 weeks of gestation, immunized infants displayed lower FEV_1_ z-scores when compared to non-immunized VLBWIs (*p* = 0.021, *p* = 0.005, Figure 3). Table S1 shows higher rates of complicated clinical courses in immunized infants (i.e., any surgeries: 32.6%, vs. 20.2%; *p* < 0.001). With regard to the neurocognitive outcome parameters, immunized VLBWIs showed lower IQs (98 vs. 100; *p* < 0.001) and higher SDQ scores (10 vs. 8; *p* < 0.001) when compared to non-immunized VLBWIs. Via multivariate regression models adjusted for group differences and known confounders, we identified severe BPD (OR 1.3; *p* = 0.006), a low gestational age (OR 0.9; *p* < 0.001), severe complications (OR 1.6; *p* < 0.001) and the decision to immunize against RSV (OR 1.9; *p* < 0.001) as clinical parameters that are associated with the caregivers’ decision to immunize VLBWIs against influenza.

From the parental perspective, influenza-immunized VLBWIs are more often characterized as chronically ill (27.1% vs. 11.6%, *p* < 0.001). They had higher rates of hospital readmissions (17.5% vs. 11.3% *p* < 0.001) and spent more nights (median of four vs. nights nights, *p* < 0.001) in the hospital in the year prior to the 6-year-follow-up.

### 3.4. Influenza Immunization Is Not Associated with Differences in Spirometry Measures or IQ/SDQ at 6 Years of Age

By stratifying the multivariate analyses to gestational age and BPD, we aimed to identify risk groups that may benefit from influenza immunization with regard to long-term lung function (Table 3) or long-term neurodevelopmental outcomes (Table 4). Contrary to our hypothesis, we observed no significant immunization-related effect on the FEV_1_ and FVC z-scores in both of the risk groups. No associations were noted between immunization and the IQ or SDQ values when adjusted for confounders.

Immunized and non-immunized infants, matched for gestational age, birth weight, gender, multiple birth, SGA and BPD by using Mahalanobis distance matching (*n* = 613 immunized infants vs. *n* = 613 non-immunized infants), were not significantly different in their FEV_1_ and FVC v z-scores (Table S2). Despite matching, immunized infants displayed more bronchitis episodes in early childhood and were characterized by heightened respiratory morbidity (tracheal ventilation 64.1 vs. 56.8%, *p* = 0.01, palivizumab administration 74.3 vs. 59.9%, *p* < 0.001). However, no differences in spirometry measures were observed between the matched groups.

### 3.5. Timing of Immunization for Peak Respiratory Vulnerability During First Influenza Season

In a subgroup analysis of VLBWIs who were at least six months old before their first influenza season—thus eligible for influenza immunization—we compared infants who received early immunization (*n* = 198) with those who were immunized later (*n* = 673). “Early immunization” was defined as vaccination before the infant’s first influenza season or within the first month of that season. Univariate analyses showed no significant differences between both groups regarding the general clinical characteristics, such as gestational age, birth weight, gender and occurrence of severe complications. The Z-scores of FEV_1_ and FVC, as well as the IQ values, did not differ between groups. Infants who received an early first influenza immunization had lower rates of bronchitis at 6 years of age than those who did not (8.2% versus 32.6%, *p* < 0.001). The multivariate analysis did not show that early immunization had an effect on the FEV_1_ and FVC z-scores; however, we observed a protective effect on the long-term bronchitis risk, even when adjusted for confounding factors and controlled for multiple testing using Bonferroni correction (bronchitis episodes in the last year before follow-up; OR: 0.19, CI: 0.07–0.55, *p* = 0.002).

## 4. Discussion

In this large population-based cohort study of GNN preterm infants, 26% of six-year-old VLBWIs were vaccinated against influenza, whereas only 12% of eligible infants were immunized before their first influenza season after hospital discharge. The key factors driving the decision to vaccinate against influenza were the presence of BPD, a low gestational age and neonatal complications. From our observational data, we cannot conclude that immunization has beneficial effects on lung function (FEV_1_ and FVC) or neurocognitive outcomes at early school age (IQ and SDQ). However, there were no safety signals observed, and early immunization against influenza to achieve protection during the first season after hospital discharge was associated with a reduced bronchitis risk at six years of age.

To our knowledge, this is the first population-based epidemiological study on influenza immunization in German VLBWIs. Compared to international data, such as the 41% to 64% immunization coverage reported in the United States by the CDC and a retrospective study from Washington state [34], we report comparatively low immunization rates in Germany. This discrepancy may be explained by German guidelines, which recommend influenza immunization only for infants with chronic conditions or immune impairments, in contrast to CDC and World Health Organization (WHO) recommendations advocating vaccination for all children aged ≥6 months [35]. Furthermore, the vaccination rates in our study reflect a minimal threshold definition (≥1 dose of the vaccine), suggesting that the actual coverage may be even less comprehensive. Low vaccination rates and various other aspects of our data point towards a lack of rigor in the influenza vaccination strategy in place. First, only 12% of eligible VLBWIs were immunized prior to their first influenza season. Second, only a few infants received annual immunizations to ensure continuous protection until school age. Finally, the vaccination rates in Germany varied significantly by region, underscoring the absence of specific national guidelines for preterm infants.

VLBWIs often experience schedule delays in receiving routine vaccinations [36]. These delays are primarily attributed to misinformation among parents and healthcare workers, who may believe that vaccinations not only cause avoidable pain but could also trigger harmful inflammatory responses that vulnerable preterm infants might not tolerate well. However, vaccine hesitancy may be even more pronounced for risk group vaccinations that lack clear guidelines. In this context, it is important to note that no safety concerns regarding early immunizations were observed in our data, but that protection against the long-term risk of bronchitis was observed. Given the heightened risk of influenza-related complications in VLBWIs, specific vaccination programs for preterm infants, like those implemented in Norway, are warranted [37]. Evidence from health registry data supports these efforts, as influenza-related hospitalizations are most common during the first year of life, particularly in infants with underlying conditions, such as BPD [38]. Our data provide evidence that BPD and a low gestational age are the main factors that drive healthcare providers to recommend influenza vaccination in VLBWIs. BPD represents a well-known marker of heightened vulnerability, as it is associated with chronic lung impairment and an increased risk of severe respiratory infections. While preterm birth itself is a known risk factor for complicated courses of influenza, including higher rates of hospitalization, longer illness durations, and greater healthcare utilization [1,6,37], it does not appear to be a standalone factor with sufficient impact to strongly influence vaccination decisions. This may reflect the general perception that preterm birth, in the absence of specific complications like BPD, does not warrant targeted immunization. However, this approach may underestimate the substantial risk influenza poses to preterm infants, including severe or even fatal infections, increased medication use, school absenteeism, and prolonged recovery compared to term infants. Notably, existing data on the safety and immunogenicity of influenza vaccines suggest comparable outcomes between preterm and full-term populations, though the efficacy of the data remains limited [39]. These findings underscore the importance of considering broader criteria, beyond specific complications, when developing vaccination strategies for this high-risk group. Additionally, our data support the hypothesis that influenza immunization might not only protect specifically against influenza, but may also promote unspecific trained immunity [40], potentially reducing the risk of infections that are not preventable by vaccination. The reduced long-term risk of bronchitis among VLBWIs immunized early may reflect these pathogen-agnostic effects. Trained immunity is characterized by immune cell programming (e.g., myeloid cells), epigenetic chromatin alterations influencing inflammatory pathways, the heightened production of proinflammatory cytokines (e.g., IFN-γ), and the non-antigen-specific activation of natural killer cells, all of which play a vital role in immunological memory [41]. Since trained immunity effects have been demonstrated for various vaccines [42] and have been hypothesized to affect preterm infants in previous studies [36,43], this information should be incorporated into general education about vaccinations to enhance awareness and understanding. This observational study underscores the importance of prioritizing immunization strategies that protect infants during their first influenza season, aligning with national guideline recommendations. To enhance vaccination coverage and timing, efforts should focus on educating healthcare providers and caregivers to address misconceptions about the safety of immunization in this vulnerable population.

Our main hypothesis was not confirmed, as this study did not find that influenza immunization had an effect on lung function or neurocognitive outcomes at six years of age. The impaired FEV_1_ and FVC z-scores in the univariate analyses may represent the increased general vulnerability of immunized infants, who are characterized by a lower gestational age and higher rates of complications. Neonatal lung damage, together with preterm birth, may play an most important role in functional respiratory long-term impairment, which is—in VLBWIs—characterized by airway obstruction, hyperinflation, and diffusion impairment [44]. However, independent vaccination-related outcome effects were not observed in the multivariate and matched pair analyses, which accounted for the most important confounders. Previous GNN data showed that seasonal RSV prophylaxis with palivizumab is likewise not associated with improved lung function at early school age [45]. However, our data must be interpreted in the complex context of the confounders that impact long-term outcomes in preterm infants. Respiratory outcomes depend on underlying pathophysiologic mechanisms, such as the prematurity-related early disruption of the alveolar and vascular development of the lungs, resulting in structural simplification and airway smooth muscle disfunction. High rates of invasive ventilation, sustained inflammation and severe infections alter respiratory outcomes, and polygenetic risk scores predispose a subgroup of infants to obstructive pulmonary disease of prematurity. Further research is needed to elucidate the complex interplay between influenza immunization, respiratory morbidity, and long-term outcomes in VLBWIs, guiding the development of evidence-based interventions to optimize health in this population.

### Strengths and Limitations

The primary strength of this study is the large cohort size and the highly standardized on-site follow-up examination at six years by a study team trained in neonatology. The main limitation of this study is the fact that it is a post hoc analysis of an observational population-based study. Consequently, it can only point out associations and no causal relationships can be proven. Furthermore, data on influenza infection and the vaccination coverage of household members and close contacts (cocooning strategy) are unavailable. Lung function assessments in preterm children are subject to selection bias, as these tests can only be performed on children who are able to understand and follow the instructions for the breathing maneuvers. In the multivariate analyses, we aimed to adjust for the most important confounders in order to minimize indication bias. Although we used regression models to account for potential confounding variables in our analysis of observational data, the possibility of bias from unknown factors cannot be entirely excluded, as adjustments cannot fully capture all the etiological aspects of preterm birth that may influence neonatal outcomes. Finallu, statistical adjustments alone may not fully capture the biological impact of key determinants on long-term lung health.

## 5. Conclusions

We conclude that clinicians tend to prioritize influenza immunization for infants born at less than 28 gestational weeks and those with significant morbidities, particularly BPD. However, our observational data show that the current practice of influenza vaccination for German VLBWIs may not provide the maximum possible benefit for this vulnerable population or that its protective effects could be enhanced with optimal implementation. Specifically, low immunization coverage, significant regional disparities, and vaccination delays leave many VLBWIs unprotected during their most vulnerable first influenza season after hospital discharge. Our data show an association between early influenza vaccination and a reduced number of bronchitis cases between the ages of 5 and 6 years, so that the hypothesis of influenza vaccination-related trained immunity effects in preterm infants is cautiously supported. Importantly, no safety concerns related to early immunization were observed, emphasizing its safety and feasibility for broader implementation in this population. Future immunization strategies should focus on timely vaccination, and the establishment of preterm infant-specific guidelines may be critical for reducing morbidity from respiratory infections and improving long-term health outcomes.

## Figures and Tables

**Table 1:** Clinical characteristics of the study cohort stratified for influenza immunization.

	Not Immunized (*n* = 2487, 74.1%)	Immunized (*n* = 871; 25.9%)	*p*	Total (*n* = 3358)
	Median [IQR]			Median [IQR]
Gestational age (weeks)	28.43 [26.71–30.0]	27.29 [25.43–29.0]	<0.001	28.14 [26.29–29.71]
Birth weight (grams)	1030 [805–1265]	900 [690–1180]	<0.001	990 [770–1240]
Age at follow-up (years)	5.75 [5.41–6.0]	5.75 [5.41–6.0]	0.754	5.75 [5.41–6.0]
	% (95%CI)			
Female	50 (48.1–52)	45.1 (41.8–48.4)	0.012	48.7 (47.1–50.4)
Multiple birth	38.3 (36.4–40.2)	40.1 (36.9–43.4)	0.336	38.8 (37.1–40.4)
SGA	14.5 (13.2–15.9)	16.9 (14.5–19.5)	0.098	15.1 (13.9–16.4)
Vaginal birth	8.4 (7.3–9.5)	11.4 (9.4–13.7)	0.008	9.2 (8.2–10.2)
Caesarean section	91.6 (90.5–92.7)	88.6 (86.3–90.5)	0.007	90.8 (98.8–91.8)
German maternal descent	82.5 (81–84)	80 (77.2–82.5)	0.092	81.9 (80.5–83.1)
Breastfeeding	66.3 (63.7–68.8)	70.1 (65.7–74.2)	0.145	67.3 (65–69.5)
Tracheal ventilation	50.1 (48.1–52)	66.6 (63.4–69.7)	<0.001	54.4 (52.7–56.1)
Heptavalent immunization	97.9 (97.2–98.4)	99.8 (99.3–100)	<0.001	98.4 (97.9–98.8)
MMR immunization	98.7 (98.2–99.1)	99.8 (99.3–100.0)	0.007	99.0 (98.6–99.3)
RSV immunization	55.9 (53.9–57.8)	76.3 (73.4–79)	<0.001	61.2 (59.5–62.8)

Legend: SGA, small for gestational age; MMR, mumps measles rubella; RSV, respiratory syncytial virus; IQR, interquartile range, CI, confidence interval; *p*-values for univariate analyses were derived from the chi-square test or Mann–Whitney U.

**Table 2:** Pulmonological and neurocognitive outcomes and risk factors of study cohort stratified to influenza immunization.

	Not Immunized (*n* = 2487, 74.1%)	Immunized (*n* = 871; 25.9%)	*p*	Total (*n* = 3358)
	% (95%CI)			
Older Siblings	27.4 (25.7–29.2)	29.5 (26.5–32.6)	0.242	28 (26.5–29.5)
Smoke Exposure	40.5 (38.4–42.6)	41.6 (38.2–45.2)	0.576	40.8 (39–42.6)
Secondary school certificate/high school diploma of mother	78.8 (76.3–81.2)	77.0 (72.5–81.1)	0.468	78.3 (76.1–80.4)
Mild BPD	36.9 (35.0–38.8)	39.4 (36.2–42.7)	<0.001	37.5 (35.9–39.2)
Severe BPD	15.8 (14.4–17.3)	29.4 (26.5–32.5)	<0.001	19.3 (18.0–20.7)
Discharge into influenza season (December–march)	33.5 (31.7–35.4)	36.0 (32.8–39.2)	0.189	34.2 (32.6–35.8)
FEV_1_ < 80%	23.6 (21.8–25.4)	32.6 (29.1–36.3)	<0.001	25.7 (24.1–27.4)
Bronchitis episodes	21.1 (19.5–22.8)	31.7 (28.6–34.9)	<0.001	23.9 (22.4–25.3)
Median (IQR)				
Invasive ventilation/CPAP (days)	28 (8–47)	41 (20–64)	<0.001	31 (10–53)
FEV_1_ (z-score)	−1.28 (−2.11–[−0.53])	−1.50 (−2.57–[−0.74])	<0.001	−1.34 (−2.17–[−0.59])
FVC (z-score)	−1.36 (−2.25–[−0.63])	−1.55 (−2.43–[−0.78])	<0.001	−1.40 (−2.34–[−0.65])
IQ	100 (92–109)	98 (88–107)	<0.001	99 (91–109)
SDQ	8 (5–12)	10 (6–14)	<0.001	9 (5–13)

Legend: FEV_1_, forced expiratory volume in one second; FVC, forced vital capacity; IQ, intelligence quotient; SDQ, strengths and difficulties questionnaire; BPD, bronchopulmonary dysplasia; IQR, interquartile range, CI, confidence interval; *p*-values for univariate analyses were derived from the chi-square test, *t*-test (IQ) or Mann–Whitney *U* test. Bronchitis episodes as reported in follow-up parental questionnaire regarding the last 12 months before 5–6-year follow-up examination.

**Table 3:** Effects of influenza immunization on FEV_1_ and FVC z-scores stratified to gestational age and BPD-defined subgroups.

Subgroup	Regression Coefficient B for FEV_1_ z-Score; (95% CI); *p*-Value	Regression Coefficient B for FVC z-Score; (95% CI); *p*-Value
22–26 *	−0.102 (−0.295–0.091) *p* = 0.300	−0.136 (−3.37–0.065) *p* = 0.183
27–28 *	−0.065 (−0.248–0.119) *p* = 0.487	−0.084 (−0.284–0.116) *p* = 0.409
29–30 *	−0.256 (−0.475–[−0.037]) *p* = 0.022 **	−0.089 (−0.317–0.140) *p* = 0.447
31–35 *	0.101 (−0.306–0.507) *p* = 0.627	0.232 (−0.190–0.655) *p* = 0.280
Mild BPD	−0.126 (−0.293–0.042) *p* = 0.142	−0.103 (−0.285–0.079) *p* = 0.266
Severe BPD	−0.065 (−0.320–0.191) *p* = 0.619	−0.142 (−0.408–0.124) *p* = 0.293

Legend: CI, confidence interval; BPD, bronchopulmonary dysplasia; FEV_1_, forced expiratory volume in one second; FVC, forced vital capacity. Linear regression models were performed using gestational age, multiple birth, tracheal ventilation, blood culture-proven sepsis, severe complications (IVH grade III or IV, periventricular leukomalacia, surgeries for retinopathy of prematurity, focal intestinal perforation, persistent ductus arteriosus or for a ventricular peritoneal shunt), SGA, IQ, bronchitis episodes, RSV immunization and influenza immunization as independent variables. * gestational age in weeks. ** does not reach statistical significance after Bonferroni correction for multiple testing.

**Table 4:** Effects of influenza immunization on IQ and SDQ, stratified to gestational age.

Subgroup	Regression Coefficient B for IQ; (95% CI); *p*-Value	Regression Coefficient B for SDQ; (95% CI); *p*-Value
22–26 *	0.013 (−2.1–2.12) *p* = 0.990	0.821 (−0.03–1.68) *p* = 0.060
27–28 *	−0.734 (−2.74–1.27) *p* = 0.473	0.887 (0.04–1.73) *p* = 0.040 **
29–30 *	0.432 (−1.88–2.75) *p* = 0.714	0.135 (−0.83–1.10) *p* = 0.783
31–35 *	−5.877 (−10.62–[−1.13]) *p* = 0.015 **	−1.049 (−3.01–0.91) *p* = 0.293

Legend: CI, confidence interval; IQ, intelligence quotient; SDQ, strengths and difficulties questionnaire. Linear regression models were created using gestational age, gender, multiple birth, blood culture-proven sepsis, severe complications (IVH grade III or IV, periventricular leukomalacia, surgeries for retinopathy of prematurity, focal intestinal perforation, persistent ductus arteriosus or for a ventricular peritoneal shunt), SGA and influenza immunization as independent variables. SDQ was included in the linear regression model for IQ as the dependent variable and vice versa. * gestational age in weeks. ** does not reach statistical significance after Bonferroni correction for multiple testing.

## Data Availability

The data that support the findings of this study are available from the corresponding author, [M.-T.D.], upon reasonable request.

## Supplementary Materials

The following supporting information can be downloaded at: https://www.mdpi.com/article/10.3390/vaccines13010042/s1, Figure S1: Influenza Vaccination Doses and Corresponding Percentages; Figure S2: Chronological Age at first Influenza Immunization by Gestational Age; Table S1: Treatment and outcome parameters stratified to influenza immunization; Table S2: Clinical characteristics and respiratory outcomes of matched VLBWI at 6-year follow-up.

## Abbreviations

BPD	Bronchopulmonary dysplasia
CDC	Centers for Disease Control and Prevention
CI	Confidence interval
CLD	Chronic lung disease
CPAP	Continuous positive airway pressure
ECDC	European Centers for Disease Control and Prevention
ELGAN	Extremely low gestational age neonates
FEV_1_	Forced expiratory volume in one second
FVC	Forced vital capacity
GDR	German Democratic Republic
GNN	German Neonatal Network
IQ	Intelligence quotient
MMR	Mumps, measles, rubella
NICU	Neonatal Intensive Care Unit
OR	Odds Ratio
RKI	Robert Koch Institute
ROP	Retinopathy of prematurity
SDQ	Strengths and difficulties questionnaire
SGA	Small for gestational age
VLBWI	Very low birth weight infants
WHO	World Health Organization
WPPSI	Wechsler Preschool and Primary Scale of Intelligence
