# Supplementary material for: Influenza Immunization in Very-Low-Birth-Weight Infants: Epidemiology and Long-Term Outcomes
Source: Vaccines (Basel). 2025 Jan 7;13(1):42. doi: 10.3390/vaccines13010042 (PMC11769184; doi:10.3390/vaccines13010042)
Supplement: Supplementary file 1 [file vaccines-13-00042-s001.zip › vaccines-3348715-supplementary.pdf]

Supplementary material

Table S1: *Treatment and outcome parameters stratified to influenza immunization.*

	**Not immunized** (*n*= 2487, 74.06%)	**Immunized** (*n*= 871; 25.94%)	**p**	**total** (*n*= 3358)
	**% (95% CI)**			
**Surgery**	20.2 (18.7 – 21.8)	32.6 (29.5 – 35.7)	<0.001	23.4 (22.0 – 24.9)
**ROP surgery**	2.9 (2.3 – 3.6)	6.0 (4.6 – 7.7)	<0.001	3.7 (3.1 – 4.3)
**PDA surgery**	2.9 (2.3 – 3.7)	7.7 (6.1 – 9.6)	<0.001	4.2 (3.5 – 4.9)
**NEC surgery**	2.1 (1.6 – 2.7)	2.9 (1.9 – 4.1)	0.183	2.3 (1.8 – 2.8)
**FIP surgery**	2.1 (1.6 – 2.8)	3.5 (2.4 – 4.8)	0.03	2.5 (2.0 – 3.0)
**VP shunt surgery**	1.2 (0.9 – 1.7)	3.9 (2.8 – 5.4)	<0.001	1.9 (1.5 – 2.4)
**IVH**	14.3 (13.0 – 15.7)	27.0 (24.1 – 30.0)	<0.001	17.6 (16.3 – 18.9)
**Blood culture-proven Sepsis**	12.2 (11.0 – 13.5)	16.6 (14.2 – 19.1)	0.001	13.3 (12.2 – 14.5)
**Severe complications**	11.7 (10.5 – 13.0)	24.8 (22.0 – 27.7)	<0.001	15.1 (13.9 – 16.3)

Legend: *ROP, retinopathy of prematurity; PDA, persistent ductus arteriosus; NEC, necrotizing enterocolitis; FIP, focal intestinal perforation; VP, ventriculoperitoneal; IVH, intraventricular hemorrhage; CI, confidence interval; p-values for univariate analyses were derived from chi-square test.*
Severe complications encompass IVH grade III or IV, periventricular leukomalacia, surgeries for retinopathy of prematurity, focal intestinal perforation, persistent ductus arteriosus or for a ventricular peritoneal shunt.

Table S2*. Clinical characteristics and respiratory outcomes of matched VLBWI at 6-year follow-up.*

	**Not immunized** (*n*= 613)	**Immunized** (*n*= 613)	**p**
	**Median [IQR]**		
**Gestational age** (weeks)	27.9 [26.3 – 29.4]	27.5 [25.7– 29.1]	0.1
**Birth weight** (grams)	960 [730 – 1210]	940 [720– 1210]	0.3
	**% (95% CI)**		
**Multiples**	39.9 (35.5 – 43.2)	41.3 (37.4 – 45.2)	0.5
**Male gender**	47.8 (43.9 – 51.8)	45.0 (41.2 – 49.0)	0.1
**SGA**	16.3 (13.6 – 19.4)	14.8 (12.2 – 17.8)	0.5
**BPD**	24.8 [21.5 – 28.3]	24.8 [21.5 – 28.3]	1.0
**Outcome**	**% (95% CI)**		
**Palivizumab**	59.9 (56.0 – 63.7)	74.3 (70.8 – 77.7)	<0.001
**Tracheal ventilation**	56.8 (52.8 – 60.7)	64.1 (60.3 – 67.8)	0.01
**FEV < 80%**	28.7 (24.9 – 32.7)	30.0 (26.0 – 34.0)	0.7
**Bronchitis episodes (year before follow-up)**	24.1 (20.8 – 27.7)	32.8 (29.1 – 36.7)	0.001
	**Median [IQR]**		
**FEV1 (z-score)**	-1.41 (-2.29 – [-0.64])	-1.46 (-2.34 – [-0.75])	0.7
**FVC (z-score)**	-1.49 (-2.45 – [-0.74])	-1.56 (-2.51 – [-0.65])	0.7
**FEV1 (l)**	1.0 (0.8 – 1.18)	1.0 (0.8 – 1.2)	0.6
**FVC (l)**	0.93 (0.8 – 1.2)	0.92 (0.8 – 1.2)	0.9

Legend: Outcome of matched cohort for influenza immunization.
Mahalanobis distance matching criteria were gestational age, birth weight, gender, multiple birth, SGA and BPD.
P-values are derived from T-Test (IQ), Pearson’s Chi-square test or Mann-Whitney U-test; the type I error level was set to 0.05; data are given as median and IQR or % and 95% confidence interval.

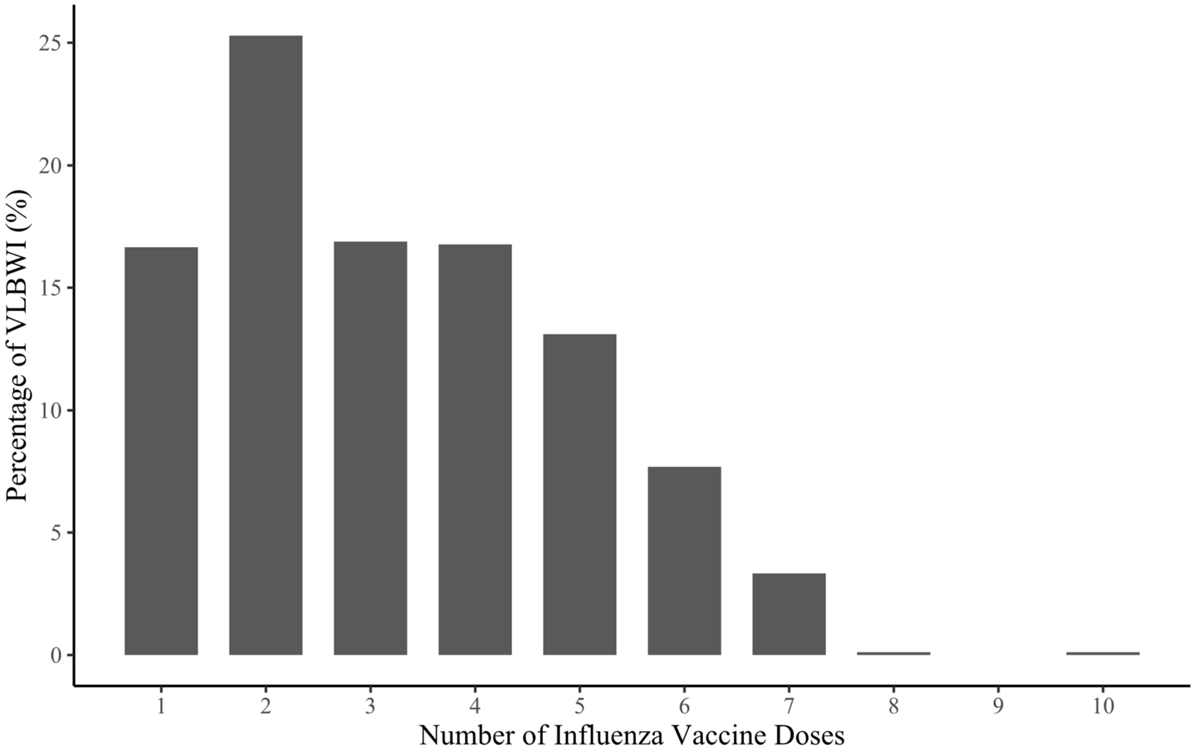

Figure S1: *Influenza Vaccination Doses and Corresponding Percentages. The bars show the number of administered influenza vaccinations and percentage of VLBWI receiving the respective number of vaccinations. A total of n=2803 doses of influenza vaccine were administered to n=871 VLBWI. Most VLBWI received two vaccine doses (25,3%), while in 16.7% of the cases only one dose was administered (three doses in 16.9%, four doses in 16.8%, five doses in 13.1% and six doses in 7.7%.*

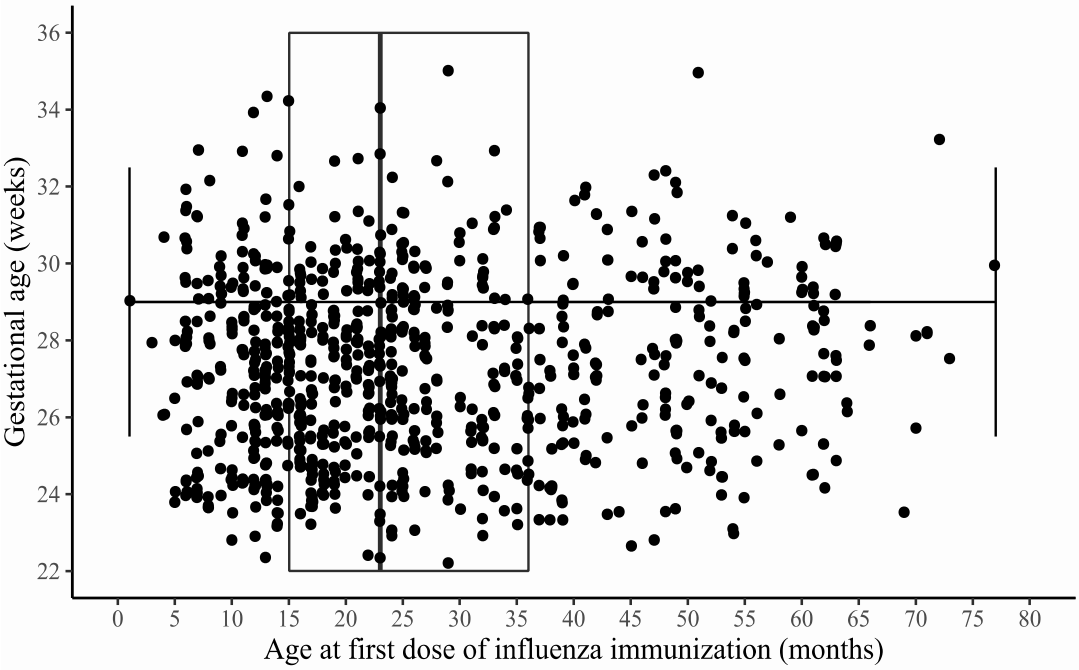

Figure S2: Chronological Age at first Influenza Immunization by Gestational Age. The dot plot shows the distribution of chronological age in months at first influenza immunization stratified to gestational age. Data points are depicted as a scatter plot. Data of age at first immunization is shown as a box plot indicating median, interquartile range, minimum and maximum.
